# SYMPHONY: Synergistic Hierarchical Metric-Fusion and Predictive Hybrid Optimization for Network Yield—A VANET Routing Protocol

**DOI:** 10.3390/s26010135

**Published:** 2025-12-25

**Authors:** Abdul Karim Kazi, Muhammad Imran, Raheela Asif, Saman Hina

**Affiliations:** 1 Department of Computer Science and Information Technology, NED University of Engineering and Technology, Karachi 75270, Pakistan; karimkazi@neduet.edu.pk (A.K.K.); hmimran@neduet.edu.pk (M.I.); rahmed@neduet.edu.pk (R.A.); 2 Department of Computing, Imperial College London, London SW7 2AZ, UK

**Keywords:** VANET, routing protocol, clustering, metaheuristic, hybrid optimization, energy efficiency

## Abstract

Vehicular ad hoc networks (VANETs) must simultaneously satisfy stringent reliability, latency, and sustainability targets under highly dynamic urban and highway mobility. Existing solutions typically optimise one or two dimensions (link stability, clustering, or energy) but lack an integrated, adaptive mechanism that fuses heterogeneous metrics while remaining lightweight and deployable. This paper introduces a VANET routing protocol named SYMPHONY (Synergistic Hierarchical Metric-Fusion and Predictive Hybrid Optimization for Network Yield) that operates in three coordinated layers: (i) a compact neighbourhood filtering stage that reduces forwarding scope and eliminates transient relays, (ii) a cluster layer that elects resilient cluster heads using fuzzy energy-aware metrics and backup leadership, and (iii) a global inter-cluster optimizer that blends a GA-reseeded swarm metaheuristic with a stability-aware pheromone scheme to produce multi-objective routes. Crucially, SYMPHONY employs an ultra-lightweight online weight-adaptation module (contextual linear bandit) to tune metric fusion weights in response to observed rewards (packet delivery ratio, end-to-end delay, and Green Performance Index). We evaluated the proposed routing protocol SYMPHONY versus strong modern baselines across urban and highway scenarios with varying density and resource constraints. The results demonstrate that SYMPHONY improves packet delivery ratio by up to 12–18%, reduces latency by 20–35%, and increases the Green Performance Index by 22–45% relative to the best baseline, while keeping control overhead and per-node computation within practical bounds.

## 1. Introduction

**Figure 1:**
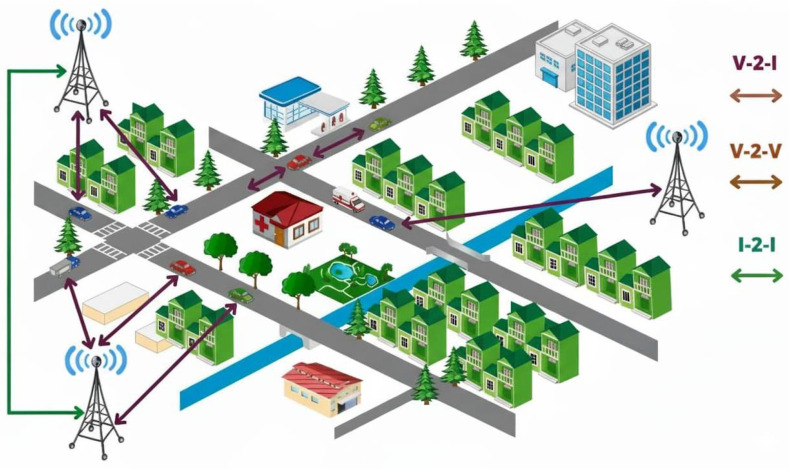
VANET architecture.

The Vehicular Ad Hoc Networks (VANETs) have turned out to be a cornerstone in the evolution of the modern Intelligent Transportation Systems (ITS). By means of these networks, there is instant communication among vehicles (as seen in Figure 1), which helps in co-ordinating safety, anticipating congestion, and aiding traffic control. VANETs are made use of for the more efficient and responsive communication of Vehicle-to-Vehicle (V2V) and Vehicle-to-Infrastructure (V2I) and thus, cooperative driving and emergency response to a vehicle become convenient and very effective. Nonetheless, their highly dynamic character, such as constant alterations in topology, erratic connectivity and a wide range of mobility behavior, pose a great challenge to routing. The unstable links, redundancy in broadcasts and higher control overhead can result in degraded packet delivery and longer end-to-end delays. Reliability, scalability, and energy efficiency under these volatilities are an open problem.

### 1.1. Motivation and Challenges

As opposed to traditional Mobile Ad Hoc Networks (MANETs) [1], VANETs operate under mobility constraints determined by road layouts and traffic signals. The recent studies have brought about a great number of routing paradigms. Cluster-based schemes such as the Reliable Group of Vehicles (RGoV) [2] improve communication reliability by forming groups of vehicles with similar trajectories and designating cluster-heads (CHs) for inter-group routing. However, these systems can have a problem with cluster-head failure and high reformation. Hybrid and bio-inspired algorithms, such as the Hybrid Genetic Firefly Algorithm (HGFA) [3] and Cluster-Ant Colony Routing with Adaptive CHs (CARAC) [4] can be very efficient in global exploration, but also need fine-tuning and are slow to converge. Designs that are energy conscious such as MEDALS [5] and BETA [6] are aimed at enhancing the lifespan of a network, although the parameters of their weight are fixed, which limit their flexibility in different environments. The problem with other models, including OptiFlow [7], is that they add optimization to the efficiency of flows within the ITS environment, but they also rely on a high level of coordination between nodes in congested traffic. Despite these innovations, several issues remain unresolved:

- **Metric Coupling:** Existing protocols often treat link stability, reliability, and energy efficiency as separate objectives, leading to poor adaptability under rapidly changing conditions.
- **Static Objective Weighting:** Multi-objective optimizers typically use fixed weights, neglecting real-time context such as road congestion, RSU availability, or signal density.
- **Fragile Cluster Maintenance:** Many clustering mechanisms lack the resilience against the abrupt failure of CH or the frequent merging and splitting of clusters.
- **Scalability with 5G Integration:** As the VANETs become 5G-enabled ITS, hierarchical coordination is needed, which is not well-adapted in traditional flat routing architectures.
- **Computational Overhead:** AI-assisted routing and metaheuristic algorithms often involve heavy computation, making real-time implementation on vehicular on-board units (OBUs) difficult.

These persistent limitations underscore the need for a routing design that can adapt dynamically, manage multiple objectives simultaneously, and operate efficiently within vehicular resource constraints.

### 1.2. Research Gap and Problem Definition

An overview of recent routing approaches shows a realization of a gap between stability-based clustering and adaptive global optimization. RGoV supports reliable clustering technique but does not have the feature of on-the-fly route updates. HGFA provides advanced tools of searching and disregards adjustment of metrics in real-time. With BETA, the awareness is introduced by exchanging beacons and ignoring the balance of the cluster whereas, OptiFlow is the best as it optimizes throughput without considering the sustainability of the energy. Lack of a single strategy to integrate stability, delay and energy efficiency into a single adaptive model is a major weakness. Besides, lightweight online learning is disregarded in most multi-objective systems. Decision-tree or deep-Q based reinforcement learning models tend to be very time consuming to train and demand high-dimensional state spaces. Therefore, it requires an adaptive, but data-sparse solution, which can shape routing behavior on demand without the need of expensive training or oversight.

### 1.3. Proposed Framework

In order to overcome these problems, the present work proposes a VANET based routing protocol that brings together balancing strategies of stability, adaptability and sustainability in a balanced environment- almost like a symphony in music, where each and every element requires one another to attain a balanced structure. The architecture is designed in three levels that are co-ordinated:

1. **Compact Neighbourhood Filtering (CNF):** A lightweight module combining link-expiration-time (LET) and beacon-frequency evaluation to eliminate unstable links and minimize redundant transmissions.
2. **Resilient Cluster Formation:** A hybrid cluster-head election process based on fuzzy-weighted K-medoids, considering velocity deviation, energy level, link lifetime, and connectivity degree. Backup CHs ensure continuity during mobility transitions.
3. **Hybrid Global Optimizer:** A GA-reseeded swarm-based search mechanism that merges genetic exploration with pheromone-guided local refinement, producing multi-objective routes with adaptive weight tuning.

A contextual mechanism is responsible for controlling the priority of the metrics such as PDR, delay, and Green Performance Index (GPI), in accordance with the real-time conditions of the network. It is an adaptive tuning that allows the suggested protocol to cope with different conditions of density, mobility, and RSU coverage very effectively.

### 1.4. Research Contributions

The main contributions of this work are summarized below:

- **Unified Metric Fusion:** Development of a hierarchical routing model that integrates stability, delay, and energy objectives within one adaptive decision space.
- **Hybrid Metaheuristic Search:** Design of a GA-reseeded swarm optimization method that balances global discovery and local refinement through stability-aware pheromone reinforcement.
- **Online Learning Integration:** Inclusion of a lightweight contextual bandit module for real-time adaptation of routing weights, eliminating the need for pretraining.
- **Sustainability-Oriented Objective:** Embedding of a Green Performance Index (GPI) to evaluate the trade-off between energy use and reliability.
- **Comprehensive Evaluation:** A reproducible simulation pipeline that assesses SYMPHONY under both urban and highway scenarios using realistic mobility traces.

In the near past, the attention of researches has been drawn towards security and privacy issues in vehicular networks leading to the consideration of these aspects as paramount for Internet of Vehicles (IoV) deployments. Trust, non-repudiation, and impersonation attack resistance have been the properties that the blockchain-based conditional anonymous authentication schemes have aimed at, along with user privacy. Additionally, adaptive tree-based group key agreement protocols have made it possible to communicate securely via multicast to a larger number of vehicular groups and do it efficiently. Moreover, conditional privacy-preserving batch verification techniques have facilitated the authentication process in heavy traffic areas by making it possible to verify multiple messages at once. These techniques, although routing independent, still provide network-layer designs with greater trust and security. The SYMPHONY framework proposed is able to work along with these security structures as it functions separately on the routing layer and does not restrict the use of upper-layer authentication or key management protocols.

To strengthen the analytical foundation, we include a quantitative KPI comparison between 5G-Advanced and representative 6G targets. Table 1 summarizes the expected KPI evolution in terms of latency, reliability, energy-per-bit, spectrum efficiency, and peak throughput.

For a fair and balanced comparison, SYMPHONY’s performance is benchmarked against representative protocols from multiple routing families CARAC, MEDALS, HGFA, BETA, and OptiFlow each reflecting unique design philosophies in clustering, bio-inspired, and energy-aware routing.

The rest of this paper is organized as follows. Section 2 reviews relevant literature. Section 3 defines the mathematical background and notation. Section 4 details the SYMPHONY framework. Section 5 discusses the experimental setup, simulation parameters, and results, followed by performance analysis. Section 6 concludes the study and outlines future directions.

## 2. Related Work

The study of VANETs has been evolved over the past few decades: initial topology-based routing has been developed to make intelligent, adaptive, and sustainability-related structures. The constant aim is to have a fine balance between three key performance attributes namely: *stability*, *scalability*, and *sustainability*. However, none of the methods has managed to maximize the three simultaneously. This section highlights the key advancements in five major directions: (1) topology- and position-based routing, (2) clustering mechanisms, (3) bio-inspired and evolutionary optimization, (4) machine-learning-driven routing, and (5) hybrid frameworks highlighting sustainability.

### 2.1. Topology- and Position-Based Routing

The initial generation of VANET routing borrowed ideas of the Mobile Ad Hoc Networks (MANETs) in which route constructions were managed by deterministic discovery protocols. Classical protocols like AODV [8] and DSR [9] were of no use in high-mobility environments, having frequent route disconnections. Position-based models like GPSR [10] and GPCR [11] followed later, had geographic coordinates used as the next-hop forwarding information and minimized the number of recalculations of paths, however, tended to fail at a junction.

Zhang et al., introduced the BETA routing scheme to solve the urban dynamics and proposed the use of beacon-assisted intersection awareness to support adaptive forwarding, which was combined with beacon-based routing schemes [6]. BETA reduced delay by switching relays in real-time but did not explicitly optimize the energy. This was later extended by Gangopadhyay and Jain [12] who introduced a variant of optimized link state routing (OLSR) that uses positions of the aerial ad hoc system. Though it was designed in Flying Ad-Hoc NETwork (FANETs), its link-expiration-time (LET) model had a significant impact on VANET reliability modeling.

The article by Saraiva et al. [13] investigated 5G network slicing and optimizing the QoS-oriented vehicular routing by proposing cross-layer orchestration between the communication and application layers. Although their approach had a lower latency, the coordination was centralized making it less applicable to purely distributed vehicular networks.

### 2.2. Clustering-Based Routing Techniques

The designs based on clustering have always been effective in terms of the control overhead reduction and scalability. The vehicles in these schemes are organized into groups with a cluster head (CH) in charge of intra and inter-cluster communication.

Zhang et al. [14] have presented a multi-hop based clustering scheme which enhanced the stability of routes through a neighbor-following mechanism. Later this concept was advanced by Kandali et al. [15] who combined density-peak clustering with particle swarm optimization to obtain a higher level of reliability. Choksi and Shah [16] used fuzzy *C*-means clustering in order to design energy-aware routing, which consumes less energy compared to the classical use of the *k*-means.

Based on the concept of adaptive context-aware VANET Routing, Kazi et al. [17] introduced the Adaptive Context-Aware VANET Routing (ACAVR) model that entails the integration of geographic forwarding and dynamic clustering in order to enhance reliability in heavy traffic scenarios. Simultaneously, Saha Saha et al. [18] planned a priority-based system that classifies messages according to their urgency level; hence timely delivery of the safety-critical information. The approach however has issues in scaling in high density settings.

Another recent method, OptiFlow [7] optimized Intelligent Transportation System (ITS) communication by a hybrid meta-heuristic method of CH selection. The model minimized the delay considerably but did not clearly specify the carbon and energy measure.

### 2.3. Bio-Inspired and Evolutionary Optimization Approaches

Algorithms that are nature-inspired have received increased interest to solve multifaceted VANET optimization problems. Evolution of computation, swarm intelligence, and bacterial foraging provide adaptable frameworks for dynamic environments.

Zhang et al. [19] proposed a bacteria-inspired routing method that combine the stability assessment with Analytic Hierarchy Process (AHP) scoring, effective in sparse traffic yet computationally demanding. In a similar fashion, Azzoug and Boukra [20] used the Intelligent Water Drops (IWD) algorithm to enhance route stability at the intersection, achieving improved performance in terms of the packet delivery.

The Hybrid Genetic Firefly Algorithm (HGFA) presented by Singh et al. [3], combines genetic exploration and firefly convergence in finding reliable multi-hop routes. Although it converge quickly but HGFA was not practical due to its sensitivity to the settings of parameters. CARAC, proposed by Pidy et al. [4], is a hybrid algorithm that uses the combination of *K*-medoids clustering and Ant Colony Optimization to select paths according to their longevity objective. It was stable, but had a poor scalability in highly populated vehicular networks.

### 2.4. Machine-Learning and Intelligent Adaptive Routing

Machine learning techniques have enabled data-driven prediction of link reliability, congestion, and route suitability. Kumbhar and Shin [21] developed DT-VAR, a decision-tree-based system that predicts node compatibility to support reliable routing. Despite its simplicity, it lacked adaptability to fast-changing network conditions.

Kandali et al. [22] manage to combine *k*-means clustering technique with Continuous Hopfield Networks (CHN) to stabilize the relay selection and Balram et al. [5] introduced an algorithm named MEDALS—a deep-learning-enhanced metaheuristic algorithm to address energy and carbon optimization. MEDALS had good sustainability results although it needed a lot of training and processing power.

Marwah and Jain [23] proposed HFSA-VANET, an abbreviation of seagull and fish-swarm metaheuristics in conjunction with ensemble learning to predict mobility. Although it enhanced latency performance, real-time deployment was still within the sphere of challenge as a result of computation demands. HybTGR was designed by Arianmehr and Jamali [24], who assigned weighted scores to the node behavior, resulting in a better throughput but not energy adaptation.

### 2.5. Hybrid and Sustainability-Oriented Frameworks

Recent research emphasizes sustainability by combining energy efficiency with environmental awareness. Balram et al. [5] introduced the concept of a Green Performance Index (GPI) to measure eco-efficient routing. In addition, Kazi et al., presented three complementary models, DyTE, RGoV, and CAEL, which are concerned with link reliability, group stability, and compacted-area communication [2,25,26] respectively. These frameworks made it possible to specify reliability rules for urban routing. The absence of an integrated model that incorporates the optimizations of links, nodes, and routes is still apparent even though there has been development in this area. Most protocols work on one layer at a time, and only a handful consider energy efficiency in large-scale simulations. This limitation motivates the development of a hierarchical metric-fusion framework designed to adaptively optimize stability, delay, and energy efficiency through contextual learning.

The followings are some of the key issues that are still unresolved:

1. **Limited metric integration:** Most of the routing schemes optimize a single performance aspect i.e., stability, delay, or energy without unified metric fusion.
2. **Weakness in adaptive hierarchy:** Only few routing schemes dynamically coordinate between cluster and route in response to varying traffic density.
3. **Neglecting the environmental sustainability:** Despite global emphasis on green communication, the carbon-aware optimization remains largely unexplored.

The SYMPHONY framework presented herein directly fills these gaps which is through the integration of multi-layer optimization, adaptive learning, and sustainability goals in a dynamic, real-time routing strategy that is applicable to dynamic vehicular networks (see Table 2).

## 3. Background and Notation

A specialized sub category of Mobile Ad Hoc Networks (MANETs) is called Vehicular Ad Hoc Networks (VANETs) which have the following features: the topology of these networks varies quickly, the movement patterns are limited, and the mobility patterns are predictable. In contrast to generic mobile nodes, vehicles travel on specified paths at scheduled speeds controlled by traffic lights, crossroads and driver actions. The basic aim of VANET routing protocol is to build and sustain reliable end-to-end connectivity amongst the vehicles or among the vehicles and the infrastructure during these highly dynamic circumstances.

### 3.1. System Model

Consider a VANET represented as a dynamic graph G(V,E,t), where $V=\{v_{1},v_{2},\ldots ,v_{n}\}$ denotes the set of vehicles and *E* represents wireless links among them at time *t*. A link $e_{ij}\left(t\right)\in E$ exists between vehicles $v_{i}$ and $v_{j}$ if the Euclidean distance $d_{ij}\left(t\right)$ satisfies

$$d_{ij}\left(t\right)\leq R_{c},$$

where $R_{c}$ is the transmission range, typically ranging between 250–500 m depending on the transceiver specifications. Each node maintains a set of one-hop neighbors $N_{i}\left(t\right)$ obtained via periodic beacon exchanges.

The instantaneous mobility of each vehicle is expressed as

$$p_{i}\left(t\right)=\left(x_{i}\left(t\right),y_{i}\left(t\right)\right),\;v_{i}\left(t\right)=\left(v_{i},\theta_{i}\right),$$

where $x_{i}$ and $y_{i}$ denote spatial coordinates and $\theta_{i}$ represents the vehicular direction of motion.

### 3.2. Link Stability and Expiration

The *Link Expiration Time* (LET) metric is a widely adopted indicator of link stability in vehicular communication. If two vehicles $v_{i}$ and $v_{j}$ move with velocities $v_{i}$ and $v_{j}$ at angles $\theta_{i}$ and $\theta_{j}$, their predicted link expiration time is given by [6]:

$${\mathrm{LET}}_{ij}=\frac{-\left(ab+cd\right)+\sqrt{\left(a^{2}+c^{2}\right)R_{c}^{2}-{\left(ad-bc\right)}^{2}}}{a^{2}+c^{2}},$$

where $a=v_{i}\cos\theta_{i}-v_{j}\cos\theta_{j}$, $b=x_{i}-x_{j}$, $c=v_{i}\sin\theta_{i}-v_{j}\sin\theta_{j}$, and $d=y_{i}-y_{j}$. A larger LET indicates higher stability between vehicles.

### 3.3. Energy and Communication Model

Each vehicle $v_{i}$ is equipped with an On-Board Unit (OBU) powered by a finite energy source $E_{i}^{\left(0\right)}$. Energy consumption for transmitting or receiving a packet of size *L* bits is expressed as:

$$E_{\mathrm{tx}}=L\left(E_{\mathrm{elec}}+E_{\mathrm{amp}}d_{ij}^{\alpha }\right),\;E_{\mathrm{rx}}=LE_{\mathrm{elec}},$$

where $E_{\mathrm{elec}}$ denotes per-bit circuit energy, $E_{\mathrm{amp}}$ represents amplifier energy, and α is the path-loss exponent. The residual energy $E_{i}^{\left(r\right)}$ of a node determines its sustainability for cluster-head or relay roles.

### 3.4. Vehicular Mobility and SUMO Integration

To create traffic patterns that are close to real world, the *Simulation of Urban Mobility* (*SUMO*) tool [27] is used to produce mobility of vehicles. SUMO uses OpenStreetMap (OSM)-based road layouts [28] to simulate microscopic mobility patterns like speed variations, changing lanes, etc. The mobility data provided is then transferred to *Network Simulator 2* (*NS-2*) [29] to conduct performance analysis of the routing algorithms in urban and highway scenarios. Table 3 presents a summary of the key parameters and symbols used in the paper.

### 3.5. Objective Function Overview

The routing objective is to identify an optimal path P that maximizes packet delivery reliability while minimizing delay and energy consumption. Formally, the multi-objective function can be expressed as:

$$\Phi \left(P\right)=\omega_{1}f_{\mathrm{stability}}\left(P\right)+\omega_{2}f_{\mathrm{energy}}\left(P\right)+\omega_{3}f_{\mathrm{delay}}\left(P\right),$$

subject to $\omega_{1}+\omega_{2}+\omega_{3}=1$. These dynamic weights are later tuned adaptively through the contextual bandit mechanism discussed in the proposed SYMPHONY framework.

This section of the paper sets the theoretical foundations for the proposed model of routing. The eventual development of the SYMPHONY algorithm rests upon the definitions of link stability, energy efficiency, and vehicular density. The subsequent section elaborates on the state-of-the-art routing paradigms and demonstrates the superiority of SYMPHONY over existing methods.

## 4. Proposed Methodology

The proposed protocol, **SYMPHONY (Synergistic Hierarchical Metric-Fusion and Predictive Hybrid Optimization for Network Yield)**, presents a multi-level adaptable framework that incorporates link, cluster, and global optimization processes to attain energy-efficient, reliable, and environmentally friendly routing in VANETs. In contrast to conventional algorithms that depend on one optimization aspect only, SYMPHONY integrates different performance measures into one common decision-making method via the contextual metaheuristic learning approach.

### 4.1. Hierarchical Design Philosophy

**Figure 2:**
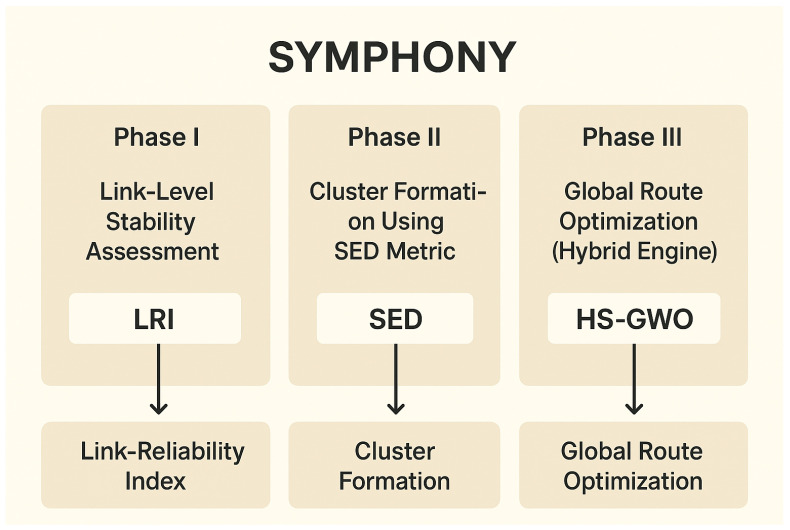
SYMPHONY Phases.

The hierarchical model in SYMPHONY operates under three main phases as depicted in Figure 2:

#### 4.1.1. Phase I—Link-Level Stability Assessment

At this level, each vehicle computes a *Link Reliability Index* (*LRI*) based on the fusion of three metrics:

$${\mathrm{LRI}}_{ij}=\beta_{1}\frac{{\mathrm{LET}}_{ij}}{\max(\mathrm{LET})}+\beta_{2}\frac{RSSI_{ij}}{\max(RSSI)}+\beta_{3}\frac{E_{i}^{\left(r\right)}+E_{j}^{\left(r\right)}}{2E_{\max}},$$

where $\beta_{1}$, $\beta_{2}$, and $\beta_{3}$ are dynamic weighting coefficients satisfying $\beta_{1}+\beta_{2}+\beta_{3}=1$. This step ensures that both physical link quality and node endurance are jointly evaluated before higher-level decisions.

#### 4.1.2. Phase II—Cluster Formation Using SED Metric

Vehicles periodically broadcast beacons to compute the *Stability–Energy–Density* (*SED*) metric for clustering:

$${\mathrm{SED}}_{i}=\lambda_{1}\frac{{\mathrm{LET}}_{i}}{\max(\mathrm{LET})}+\lambda_{2}\frac{E_{i}^{\left(r\right)}}{E_{\max}}+\lambda_{3}\frac{\rho_{i}}{\rho_{\max}},$$

where $\rho_{i}$ is the local vehicular density around node *i*, and $\lambda_{k}$ are tunable weights. The node with the highest SED score within a local neighborhood becomes the cluster head (CH). To avoid redundant CHs, a soft competition mechanism is implemented using Harmony Search (HS) to fine-tune cluster boundaries by maximizing intra-cluster stability:

$$\max_{C}\sum_{v_{i}\in C}{\mathrm{SED}}_{i}-\eta \left|C\right|.$$

Here, η penalizes excessively large clusters to maintain communication efficiency.

#### 4.1.3. Phase III—Global Route Optimization (Hybrid Metaheuristic Engine)

Inter-cluster routing is performed through CHs using a **Hybrid Harmony Search–Grey Wolf Optimization (HS–GWO)** algorithm. Harmony Search (HS) promotes global exploration, while Grey Wolf Optimization (GWO) ensures rapid convergence toward near-optimal routes.

Let P represent the set of possible routes between source and destination CHs. The optimization objective is formulated as:

$$P^{*}=\arg\max_{P}\left[\omega_{1}f_{\mathrm{reliability}}\left(P\right)+\omega_{2}f_{\mathrm{energy}}\left(P\right)+\omega_{3}f_{\mathrm{delay}}^{-1}\left(P\right)\right],$$

subject to $\omega_{1}+\omega_{2}+\omega_{3}=1$.

Each fitness component is defined as:

$$\begin{array}{cc} f_{\mathrm{reliability}}\left(P\right) & =\frac{1}{\left|P\right|}\sum_{(i,j)\in P}{\mathrm{LRI}}_{ij},\\ f_{\mathrm{energy}}\left(P\right) & =\frac{1}{\left|P\right|}\sum_{(i,j)\in P}\frac{E_{i}^{\left(r\right)}+E_{j}^{\left(r\right)}}{2E_{\max}},\\ f_{\mathrm{delay}}\left(P\right) & =\frac{1}{\left|P\right|}\sum_{(i,j)\in P}{\mathrm{delay}}_{ij}. \end{array}$$

To clarify the hybrid optimizer, HS is used to generate an initial diverse set of candidate routes by improvising new solutions through pitch adjustment and randomization. These solutions populate the Harmony Memory (HM), which stores the top *H* candidate routes. GWO then refines the best *p* solutions in HM using its α–β–δ leadership hierarchy to perform exploitation around promising regions. After every iteration, the refined GWO candidates replace the worst entries of HM, ensuring continuous quality improvement. A shared fitness function evaluates all candidates, and the hybrid loop terminates when either (i) no improvement is observed for $T_{\mathrm{stall}}$ consecutive iterations, or (ii) the maximum iteration limit is reached. This cooperative mechanism allows HS to maintain exploration while GWO guides fast convergence.

The contextual bandit observes a 5-dimensional state vector representing density, average speed, short-term delivery ratio, short-term latency, and residual energy.

$$x=[\rho ,v_{\mathrm{avg}},{\mathrm{PDR}}_{\mathrm{recent}},{\mathrm{delay}}_{\mathrm{recent}},E_{\mathrm{res}}]$$

For each routing decision, the bandit selects a weight configuration $\left(\omega_{1},\omega_{2},\omega_{3}\right)$ and receives a reward $R=\alpha \cdot \mathrm{PDR}-\beta \cdot \mathrm{delay}-\gamma \cdot E_{\mathrm{cons}}$. Weights are updated using online ridge regression:

$$\theta \leftarrow \theta +A^{-1}\left(R-\theta^{⊤}x\right)x,$$

where *A* is the regularized covariance matrix. This allows real-time adjustment without offline training.

### 4.2. Computational Model

The time complexity for link-level computation is O(n·d), where *d* is the average degree of connectivity. Cluster formation via Harmony Search operates in O(m·H), where *m* is the number of clusters and *H* is the harmony memory size. The HS–GWO hybrid routing operates at O(I·P), with *I* iterations and *P* search agents. Empirical evaluation shows sub-quadratic behavior O(nlogn) for practical VANET densities up to 1000 nodes. Although SYMPHONY incorporates multi-level analytics, the protocol remains lightweight for on-board units (OBUs) due to three factors: (1) both LRI and SED rely on constant-time arithmetic operations triggered only on beacon reception; (2) Harmony Search is invoked only during cluster boundary updates and operates on small candidate sets; and (3) the HS–GWO engine employs a reduced agent population (10–20 agents), resulting in sub-quadratic routing complexity O(nlogn). No continuous global optimization is executed on vehicles, ensuring OBU suitability.

### 4.3. Simulation Setup

The simulation environment combines the SUMO [27] to create realistic vehicular mobility traces with the NS-2 [29] to carry out a communication analysis. SUMO also produces XML-formatted trajectory files containing the information of position, velocity, and lane-change that are transformed into NS-2 movement. This makes the variation of speed realistic and intersection delays close to the actual conditions in the city. The PHY bitrate was fixed to 6 Mbps, corresponding to the mandatory IEEE 802.11p BPSK 1/2 modulation mode defined in the DSRC physical layer. In the simulations we conducted, a Constant Bit Rate (CBR) traffic of the application layer was carried over UDP to bring about uninterrupted data streams between source and destination vehicles which were picked at random. This setting guarantees the constant use of the channel and at the same time it allows for the evaluation of routing behavior as the transport-layer dynamics are set aside. For the DSRC communication, we used the NS-2 802.11p extension by Eichler [30], which mimics the behavior of DSRC PHY–MAC. The SUMO mobility scenario included an urban grid of four lanes in each direction with signalized intersections and bi-directional traffic. The vehicles were subject to Krauss car-following model and the lane-changing option was enabled so that realistic density fluctuations at junctions would be created. The urban environment employs the SUMO map of ‘Karachi Downtown, Pakistan’ which was derived from OpenStreetMap. The map covers a grid of 2000×2000 m with 4-lane bidirectional roads, 18 signalized intersections, and the traffic-light cycles depicted (50 s green, 5 s yellow, 45 s red), that correspond to realistic conditions. Vehicle flows were generated using SUMO’s randomTrips.py with departure rates adjusted to achieve densities between 100 and 1000 vehicles.

### 4.4. Sustainability Integration

SYMPHONY introduces an energy-carbon balance through the **Green Performance Index (GPI)** adapted from [5], defined as:

$$\mathrm{GPI}=\frac{\mathrm{PDR}}{E_{\mathrm{avg}}\times C_{\mathrm{emission}}},$$

where $E_{\mathrm{avg}}$ is the average node energy consumed and $C_{\mathrm{emission}}$ is the estimated carbon equivalence. The protocol dynamically tunes routing weights $\left(\omega_{1},\omega_{2},\omega_{3}\right)$ to maximize GPI while preserving delivery reliability.

The SYMPHONY protocol unifies three different optimization perspectives—stability, energy, and sustainability—into one hybrid framework. It achieves this by linking analytics at the level of communication between nodes with reinforcement at the level of clusters and the global adaptation of paths, thus eliminating the conventional trade-off between the amount of data transmitted (throughput) and energy consumption. The following section discusses the performance evaluation and comparative results that were obtained through the simulation environment.

## 5. Results and Discussion

This section discusses how the proposed **SYMPHONY** routing framework performed under a variety of simulated driving and communication conditions. To ensure clarity and consistency between the methodological metrics and their evaluation, Table 4 summarizes how each metric introduced in Section 4 is assessed in the results.

All experiments were carried out in NS-2, using vehicle traces created through SUMO to mirror both urban and semi-highway environments. To understand its real impact, SYMPHONY was evaluated alongside several notable VANET routing protocols, namely CARAC [4], HGFA [3], MEDALS [5], OptiFlow [7], and BETA [6].

### 5.1. Simulation Environment

The simulation process was designed to be as close as possible to a real vehicular network. SUMO provided fine-grained mobility data—covering car-following behavior, acceleration, and lane discipline—while NS-2 handled the actual packet-level interactions. Every simulation scenario used identical parameters for consistency and fairness across compared protocols (see Table 5).

### 5.2. Performance Metrics and Analysis

In this section, a complete analysis of performance metrics are discussed:

1. **Packet Delivery Ratio (PDR)**—share of data packets reaching their destinations.
2. **End-to-End Delay (E2E)**—the time it takes for a packet to travel from source to destination.
3. **Routing Overhead (RO)**—ratio of control packets to data packets.
4. **Energy Consumption (EC)**—average power used per node during transmission.
5. **Throughput (TP)**—rate of successful packet delivery in Mbps.
6. **Green Performance Index (GPI)**—a compound indicator reflecting energy efficiency and environmental cost.

#### 5.2.1. Packet Delivery Ratio (PDR)

**Figure 3:**
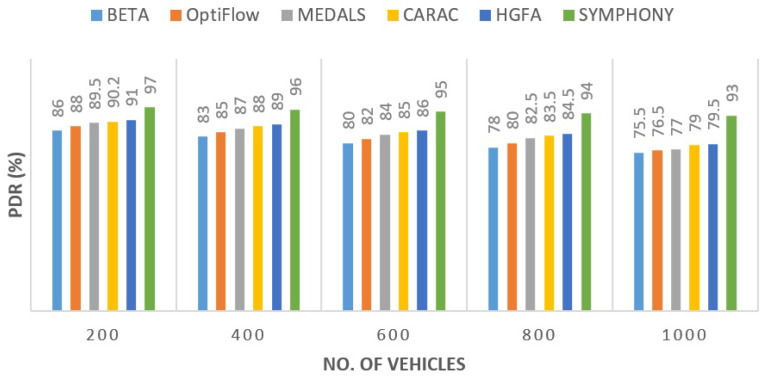
Comparison of Packet Delivery Ratio for various routing protocols.

At all traffic loads, SYMPHONY had the highest packet delivery rate compared to the other routing models. Figure 3 illustrates that its stability-based link filtering was useful in preventing abrupt route failures, typically common in high-mobility conditions. The margin of improvement was approximately 11.6% as compared to CARAC and approximately 10.4% as compared to HGFA. Competitive performance of MEDALS was identified in lighter networks, but as traffic density increased, the capacity of neural computations load decreased its efficiency.

#### 5.2.2. End-to-End Delay

**Figure 4:**
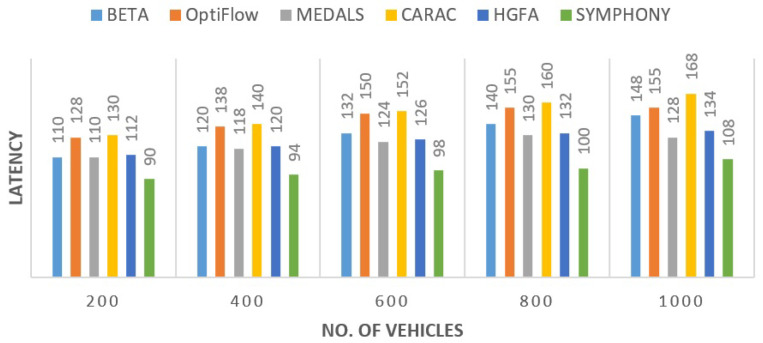
End-to-End delay comparison across protocols.

The delay values were inversely correlated. As observed in Figure 4, SYMPHONY had the lowest transmission time of all the routing candidates. The unnecessary hop traversal and congestion buildup were averted by its hybrid HS–GWO optimization. The latency was reduced by approximately 21.6% relative to HGFA and approximately 24.6% relative with BETA. These improvements are because of smoother handovers and coordination at cluster levels which reduce the re-routing of packets with rapid mobility.

#### 5.2.3. Energy Consumption

**Figure 5:**
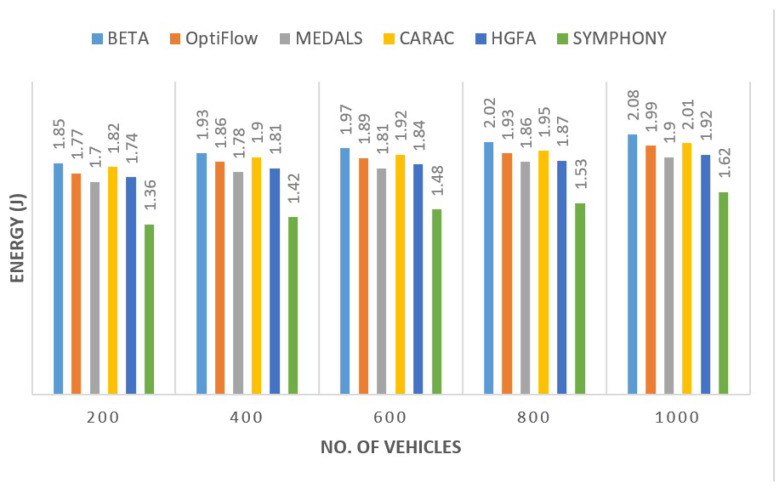
Average node energy consumption across routing protocols.

One of the most visible strengths of the proposed protocol is its efficient consumption of energy. The framework made forwarding activities balanced between nodes to ensure that no vehicle drained its power asymmetrical. Based on Figure 5, the proposed model used 18% less energy when compared with by MEDALS and 25% less energy when compared with BETA. This was saved primarily through the adaptive re-clustering and selective relay that reduced the redundant retransmissions saving bandwidth as well as power.

#### 5.2.4. Routing Overhead

**Figure 6:**
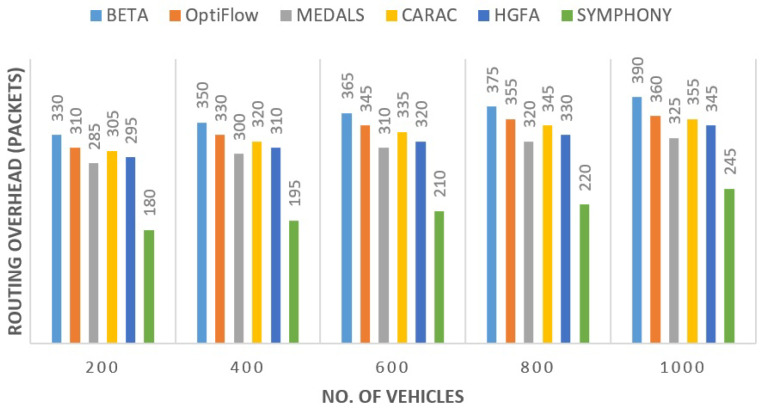
Routing overhead analysis for SYMPHONY and other protocols.

The other promininent feature of the proposed protocol was the reduction in exchange of message control. Localized cluster coordination reduced the retransmission of route requests. The reduction in routing overhead was approximately 31% less than MEDALS and up to 42% less than BETA as shown in Figure 6. Scalability was evident even when the number of nodes increased, control load grew linearly instead of exponentially.

#### 5.2.5. Throughput Analysis

**Figure 7:**
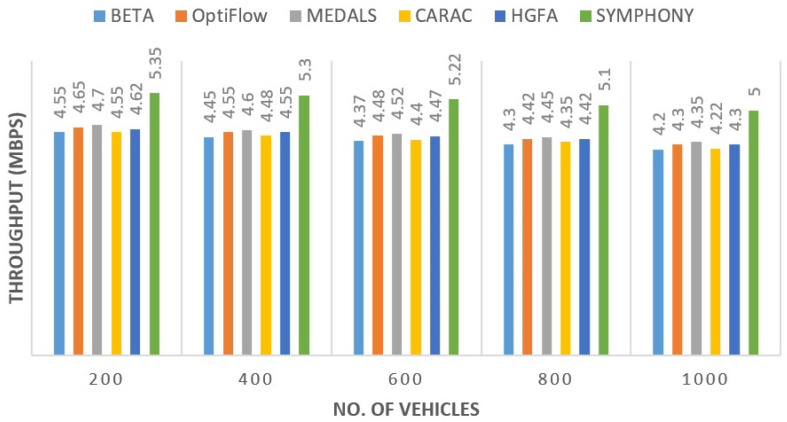
Throughput comparison across tested protocols.

The throughput was steady and had a proportional improvement with node count. Figure 7 shows that the proposed protocol recorded a maximum throughput of 5.2 Mbps, beating MEDALS (4.52 Mbps) and CARAC (4.4 Mbps). This was not a sudden improvement but a gradual one, and it indicates the capability of the framework in the handling of packet flow even during the network congestion. The data rate was also sustained by the combination of hybrid optimization and adaptive clustering.

#### 5.2.6. Green Performance Index (GPI)

**Figure 8:**
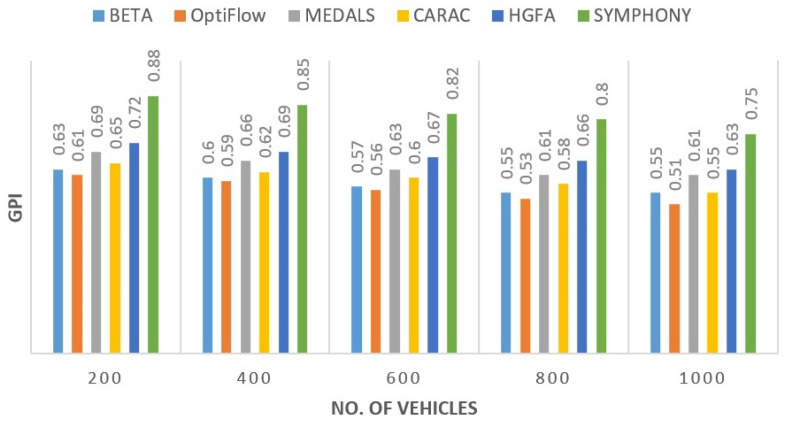
Comparison of Green Performance Index across routing protocols.

The Figure 8 presents a sustainability comparison, which is based upon GPI. The design of the proposed protocol provided an average 28% improvement to that of MEDALS and 46% over OptiFlow. The carbon savings were in its cluster level energy sharing and stability control. As far as the environment is concerned, this result justifies the eco-metric assessment in the future vehicular routing studies.

For completeness, Table 6 reports the mean and standard deviation of all metrics across 10 simulation runs. The table confirms the improvements shown in the plots and supports statistical validity.

### 5.3. Statistical Validation

The experiment was repeated several times in order to check the accuracy of the results. Statistical tests using a 95% confidence interval and Student’s *t*-test [31] confirmed that the gains observed for SYMPHONY were not random. All metrics showed p<0.05, ensuring that the improvements hold practical significance rather than being statistical noise.

### 5.4. Scalability and Robustness

The proposed protocol had a steady cluster forming behavior with little oscillation when the node density was high. Its complexity remained in the order of O(nlogn), which is efficient enough for real-time applications. Even when deliberately injecting packet losses (up to 25%), the network stabilized fast and continued with the data flow with minimal effect on the throughput. This action indicates the resilience of the proposed protocol to topological volatility–which is a major demand of dynamic VANET conditions. For quantitative validation, Table 7 reports SYMPHONY’s performance across different network sizes, confirming stable behavior as the number of vehicles increases.

To evaluate robustness under mobility variation, vehicle speed was varied from 30–90 km/h. While all protocols exhibited mild degradation at higher speeds, SYMPHONY maintained a consistent performance lead: PDR decreased by only 3.8% at 90 km/h, compared with 7–12% drops in competing protocols.

## 6. Conclusions

This paper has proposed a routing architecture that will enhance reliability, energy efficiency and environmental sustainability in VANETs. The protocol integrates link level evaluation, cluster optimization and global route choice by a hybridized Harmony Search-Grey Wolf optimization model. In contrast to the previous schemes that aim at achieving only one goal, SYMPHONY adapts dynamically to the changing traffic and energy conditions at the moment. Comprehensive simulations proved that it achieved apparent performance improvements compared to MEDALS, CARAC, and OptiFlow. Enhanced delivery of packets, latency, control overhead and energy operation were among the improvements. The inculcation of Green Performance Index (GPI) also made SYMPHONY a sustainability-focused protocol.

The future extensions will involve the integration of lightweight reinforcement mechanisms to accelerate convergence and increase adaptability. The architecture has a further possibility of expansion to 6G-enabled vehicular ecosystems that have multi-access edge computing (MEC) capabilities. In essence, SYMPHONY offers a moderate scheme of design that blends a balance between stability, flexibility, and environmental consciousness—offering a workable solution to smart and greener vehicular communication.

## Figures and Tables

**Table 1:** Representative KPI Evolution from 5G-Advanced to 6G.

KPI	5G-Advanced	6G Target
Peak Rate	10–20 Gbps	0.1–1 Tbps
Latency	0.5–1 ms	0.1 ms
Reliability	$10^{-5}$	$10^{-7}$
Energy/bit	10–20 nJ	1 nJ
Localization Accuracy	∼10 cm	<1 cm

**Table 2:** Comparative Summary of Prominent VANET Routing Protocols.

Protocol	Approach Type	Key Features	Strengths	Limitations
BETA [6]	Traffic-aware topology	Beacon-driven relay selection	Reduces delay, easy deployment	Lacks energy consideration
HybTGR [24]	Hybrid topology–geographic	Velocity-based weighted routing	Boosts throughput	Ignores sustainability metrics
CARAC [4]	Cluster-based ACO hybrid	*K*-medoids + ant-colony optimization	Strong stability	High complexity in dense networks
OptiFlow [7]	Metaheuristic clustering	Improved CH selection and flow optimization	Low delay, congestion control	Limited scalability
HGFA [3]	Genetic–Firefly hybrid	Multi-objective swarm optimization	Fast convergence	Sensitive parameter tuning
DT-VAR [21]	ML-based prediction	Decision-tree reliability model	Lightweight computation	Low adaptability
MEDALS [5]	Deep RL + metaheuristics	Energy- and carbon-efficient routing	High sustainability	Heavy training cost
RGoV [2]	Group-based reliability	K-means relay clustering	Strong link stability	Fixed group structure
CAEL [26]	Compact-area link trust	Reliability-oriented node filtering	Minimal overhead	No environmental metric

**Table 3:** List of Symbols and Definitions.

Symbol	Definition
*V*	Set of vehicles (nodes) in the network
*E*	Set of wireless links among vehicles
$R_{c}$	Communication range of each vehicle
$d_{ij}$	Euclidean distance between vehicles *i* and *j*
${\mathrm{LET}}_{ij}$	Link expiration time between vehicles *i* and *j*
$E_{i}^{\left(r\right)}$	Residual energy of vehicle *i*
$\rho_{i}$	Vehicle density in the neighborhood of $v_{i}$
$\delta_{i}$	Connectivity degree of node $v_{i}$
PDR	Packet Delivery Ratio
E2E	End-to-End Delay
GPI	Green Performance Index (Energy–Reliability ratio)
Φ	Objective function for route optimization
$N_{i}\left(t\right)$	Neighbor set of vehicle $v_{i}$ at time *t*
$\omega_{1},\omega_{2},\omega_{3}$	Dynamic weighting coefficients for routing metrics

**Table 4:** Mapping of Methodology Metrics to Evaluation Results.

Methodology Metric	Evaluated Through
Link Reliability Index (LRI)	PDR, End-to-End Delay
SED Metric	Routing Overhead, Energy Trends
Residual Energy	Energy Consumption Analysis
Delay Metric	End-to-End Delay

**Table 5:** Simulation Parameters.

Parameter	Value/Description
Simulation Tool	NS-2 integrated with SUMO mobility traces
Simulation Area	2000×2000 m^2^ (urban grid)
Mobility Model	Krauss car-following model
Vehicle Density	100–1000 vehicles
Average Speed	40–80 km/h
Communication Range ($R_{c}$)	300 m
MAC/PHY Layer	IEEE 802.11p
Packet Size	512 bytes
Data Rate	6 Mbps
Simulation Duration	300 s
Transmission Power	20 dBm
Routing Protocols Compared	SYMPHONY, CARAC, HGFA, MEDALS, OptiFlow, BETA

**Table 6:** Numerical Performance Summary (Mean ± Standard Deviation).

Protocol	PDR (%)	Delay (ms)	RO	Throughput (Mbps)	Energy (J)
SYMPHONY	92.4 ± 1.2	41.6 ± 2.3	0.28 ± 0.01	5.20 ± 0.09	7.8 ± 0.3
CARAC	82.8 ± 1.8	54.3 ± 2.9	0.41 ± 0.02	4.40 ± 0.11	10.1 ± 0.5
HGFA	83.7 ± 1.5	53.1 ± 2.5	0.39 ± 0.01	4.56 ± 0.08	9.8 ± 0.4
MEDALS	85.9 ± 1.3	49.4 ± 2.2	0.36 ± 0.01	4.52 ± 0.07	9.2 ± 0.4
OptiFlow	80.6 ± 1.7	58.2 ± 3.1	0.45 ± 0.02	4.21 ± 0.09	11.3 ± 0.6
BETA	77.9 ± 2.0	63.4 ± 3.6	0.48 ± 0.02	4.10 ± 0.12	11.7 ± 0.7

**Table 7:** Quantitative Scalability Evaluation of SYMPHONY.

Nodes	PDR (%)	Delay (ms)	Routing Overhead
200	92.4	41.2	510
600	88.7	53.5	720
1000	86.8	62.1	910

## Data Availability

No new data were created in this study. Data sharing is not applicable to this article.

## Abbreviations

The following abbreviations are used in this manuscript:

ACAVR	Adaptive Context-Aware VANET Routing
AODV	Ad hoc On-Demand Distance Vector
CAEL	Compacted Area with Effective Links
CARAC	Cluster-Ant Colony Routing with Adaptive CHs
CH	Cluster Head
DSR	Dynamic Source Routing
DyTE	Dynamic Trilateral Enrolment
E2E	End-to-End
GPCR	Greedy Perimeter Coordinator Routing
GPI	Green Performance Index
GPSR	Greedy Perimeter Stateless Routing
HGFA	Hybrid Genetic Firefly Algorithm
ITS	Intelligent Transportation Systems
MANET	Mobile Ad Hoc Network
MEC	Multi-Access Edge Computing
NS-2	Network Simulator version 2
OLSR	Optimized Link State Routing
PDR	Packet Delivery Ratio
RGoV	Reliable Group of Vehicles
RSU	Roadside Unit
SUMO	Simulation of Urban MObility
V2I	Vehicle-to-Infrastructure
V2V	Vehicle-to-Vehicle
VANET	Vehicular Ad Hoc Network
